# Early implementation of exercise to facilitate recovery after elective colorectal surgery—a randomized clinical pilot trial

**DOI:** 10.1186/s13741-026-00752-8

**Published:** 2026-10-16

**Authors:** Maike Hermann, Lisa Kusma, Niki Taebi, Tabea Kluge, Carina Mueller, Vanessa Orth, Maximilian Moeller, Carolin Ecker, Svetlana Hetjens, Julia Hardt, Christoph Reissfelder, Steffen Seyfried

**Affiliations:** 1 Department of Surgery, Medical Faculty Mannheim, University Medical Center Mannheim, Heidelberg University, Mannheim, 68167 Germany; 2 Department of Physical Therapy, Medical Faculty Mannheim, University Medical Center Mannheim, Heidelberg University, Mannheim, 68167 Germany; 3 Department of Medical Statistics and Biomathematics, Medical Faculty Mannheim, Heidelberg University, Mannheim, 68167 Germany

**Keywords:** Colorectal surgery, Colorectal cancer, Personalized medicine, Physical therapy, ERAS

## Abstract

**Background:**

Early mobilization is a key component of enhanced recovery after elective colorectal surgery. This pilot trial evaluated the safety and feasibility of an early supervised postoperative exercise intervention in addition to standard care.

**Methods:**

In this single-center randomized controlled pilot trial, 31 patients undergoing elective colorectal surgery were randomized to an intervention group (*n* = 16) or control group (*n* = 15). One patient withdrew after randomization, leaving 30 patients for modified intention-to-treat analysis. The intervention consisted of individualized, physiotherapist-supervised exercise sessions on postoperative days 1, 3, and 5. The control group received standard postoperative care according to the institutional ERAS protocol. The originally registered primary endpoint was time to first bowel movement; prior to final analysis, safety and feasibility were designated as the primary outcomes in accordance with the pilot nature of the study. The change was documented transparently. Secondary outcomes included physical performance (Short Physical Performance Battery [SPPB], handgrip strength), time to first bowel movement, patient-reported outcomes, and postoperative complications.

**Results:**

Thirty patients completed the study (retention 96.8%). Adherence to the intervention was 100%, and no intervention-related adverse events occurred. The intervention group showed significantly better preservation of physical performance, reflected by a more favorable change in SPPB score from baseline to postoperative assessment. No significant between-group differences were observed for time to first bowel movement, handgrip strength, postoperative complications, or other secondary outcomes.

**Conclusions:**

Early supervised postoperative exercise after elective colorectal surgery was safe and feasible. Although no differences were found for most secondary outcomes, the intervention improved postoperative physical performance. These exploratory findings warrant confirmation in adequately powered randomized trials.

**Trial registration:**

German Clinical Trials Register, DRKS00036327 (retrospectively registered, 22.04.2025).

**Graphical Abstract:**

**Figure:**
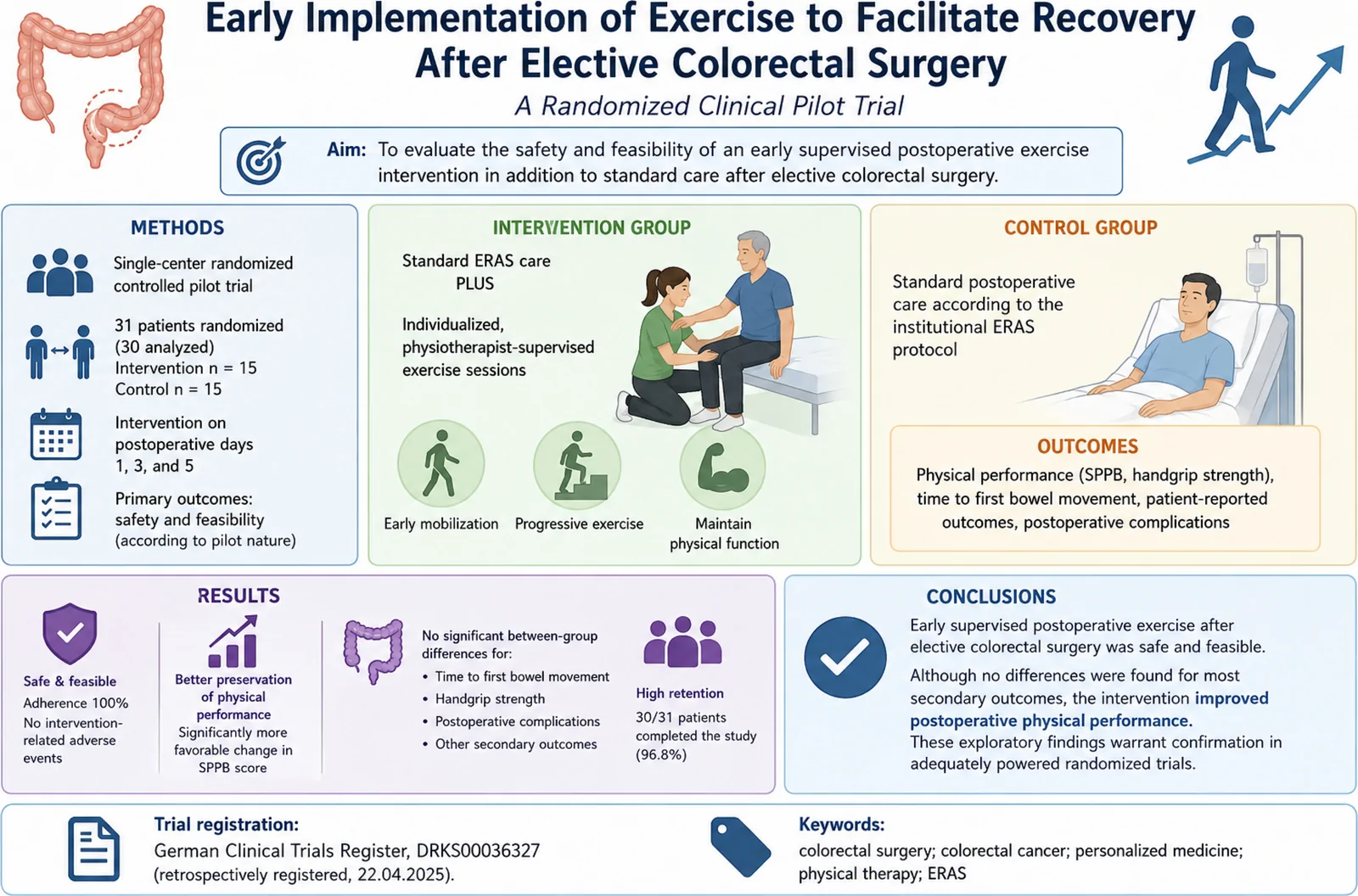

**Supplementary Information:**

The online version contains supplementary material available at https://doi.org/10.1186/s13741-026-00752-8.

## Introduction

Enhanced recovery after surgery (ERAS) pathways have established early mobilization and early oral intake as key components of perioperative care after colorectal surgery. However, the content, intensity, and implementation of postoperative mobilization vary between institutions, and the evidence for structured, individually dosed exercise interventions remains heterogeneous (Willner et al. 2023).

Postoperative exercise may help preserve physical function and facilitate recovery after colorectal surgery. Randomized trials have reported potential benefits of supervised inpatient exercise on outcomes such as length of hospital stay, readiness for discharge, and physical function, but intervention protocols, timing, supervision, and outcome measures differ substantially between studies (Gustafsson et al. 2019; Cavallaro and Bordeianou 2019). Recent systematic reviews likewise indicate potential benefits of postoperative or perioperative exercise, while emphasizing heterogeneity and the need for further adequately designed trials (Willner et al. 2023).

The present pilot randomized controlled trial therefore primarily aimed to evaluate the feasibility and safety of an early, individualized, physiotherapist-supervised postoperative exercise intervention as an adjunct to standard ERAS-based care following elective colorectal surgery. Secondary and exploratory objectives were to assess its potential effects on postoperative physical performance, gastrointestinal recovery, handgrip strength, quality of recovery, quality of life, postoperative complications, and length of hospital stay. We hypothesized that the intervention would be feasible and safe and might attenuate postoperative decline in physical performance.

## Methods

### Study design and patients

A 2-armed randomized controlled clinical pilot trial at the Department of Surgery, University Hospital Medical Center Mannheim, Germany was conducted between November 2024 (first patient in) and September 2025 (last patient out). Study eligibility included patients of either gender of full age, providing written informed consent, with benign or malignant disease limited to the colorectum undergoing elective colorectal surgery. The Ethics Committee II of Heidelberg University approved the trial.

The primary outcome, feasibility and safety of the intervention, was assessed continuously. The intervention was delivered on postoperative days 1, 3, and 5. Further, an instruction manual was provided for continuous self-training after discharge. As surrogate parameters handgrip strength, performance in the short physical performance battery test (SPPB) and on Borg-scale was measured preoperatively, on postoperative day 3. Quality of life and recovery were assessed after postoperative day 30. Secondary outcomes, such as postoperative complications on the basis of Clavien-Dindo or time to first bowel movement in [min], were monitored until postoperative day 30.

Patients and members of the public were not involved in the design, conduct, reporting, or dissemination of this pilot trial. The study was conducted in a hospital inpatient setting of a surgical department of a tertiary university hospital in Germany.

### Recruitment

Eligible patients were screened consecutively from the elective colorectal surgery schedule. Potentially eligible patients were approached by the doctoral researcher, the surgeon and study team during the preoperative assessment. Study information was provided by the doctoral researcher, inclusion was performed by a surgeon of the study team.

#### Inclusion criteria

- Age ≥ 18 years, irrespective of sex
- Diagnosis of a colorectal disease with an indication for surgical resection
- Surgical treatment at University Medical Center Mannheim
- Written informed consent provided by the participant after receiving appropriate information about the study
- Physical ability to perform cycling ergometry, treadmill exercise, or cross-trainer exercise

#### Exclusion criteria

- Patients with severe cardiac or pulmonary impairment
- Physical limitations preventing ergometry on a bicycle ergometer
- Lack of informed consent or lack of capacity to provide informed consent
- Language barrier
- Expected non-compliance
- Unresectable colorectal cancer or palliative procedures
- Pregnancy or breastfeeding

### Randomization

Participants were randomly allocated in a 1:1 ratio to either the supervised exercise intervention or standard postoperative care. The randomization sequence was generated by an independent statistician using computer-generated permuted block randomization and was provided to the study team as a password-protected Excel file. Eligible participants were assigned sequentially according to the next available allocation on the randomization list. The group allocation was accessible only to the personnel responsible for delivering the intervention and was not disclosed to other members of the study team. Due to the nature of the intervention, blinding of participants and intervention providers was not possible.

Randomization was centrally managed, and the allocation list was stored in a secure, access-restricted file. Investigators responsible for enrollment had no access to the sequence. Allocation concealment was ensured by revealing group assignment only after patient inclusion and registration.

All randomization procedures were implemented according to a prespecified protocol prior to patient enrollment.

### Intervention and postoperative care

The intervention was performed by the doctoral researcher under the supervision of a physiotherapist. Because participants and intervention providers could not be blinded, performance and observer bias cannot be excluded.

All participants received standard perioperative care according to the local ERAS protocol at University Medical Center Mannheim. The local ERAS pathway was based entirely on the current German POMGAT guideline and included standardized perioperative measures for patients undergoing colorectal surgery. These included optimized preoperative preparation, early postoperative mobilization, early oral nutrition, multimodal analgesia, and standardized postoperative monitoring and management. The ERAS protocol was applied equally to participants in both study groups (Post and Vilz 2023).

The intervention therefore did not replace routine postoperative mobilization but consisted of an additional structured, physiotherapist-supervised exercise intervention. In contrast to routine mobilization, the intervention involved a predefined aerobic exercise modality, individualized exercise intensity, physiological monitoring, and standardized supervision. Patients allocated to the intervention group underwent supervised exercise sessions on postoperative days 1, 3, and 5.

Prior to each intervention session, patients were assessed clinically and vital parameters, including blood pressure, heart rate, and oxygen saturation, were checked. Exercise modality and workload were individualized according to the patient's age, preoperative functional status, previous exercise experience, and postoperative clinical condition. The exercise modality was either a cycle ergometer or a cross-trainer. Allocation to cycle ergometer versus cross-trainer was based on a structured assessment by the doctoral researcher and supervising physiotherapist, considering preoperative fitness, age, comorbidities, and patient comfort. Patients were broadly categorized as older/untrained beginners or younger/fitter patients to guide initial protocol selection.

For older or untrained beginners using the cycle ergometer, the initial protocol consisted of 5 min at 25 W, followed by a recovery interval, 5–10 min at 50 W, a second recovery interval, and a further 5 min at 25 W. For younger or fitter patients, the corresponding protocol consisted of 5 min at 50 W, followed by 5–10 min at 75–100 W and a further 5 min at 50 W.

For older or untrained beginners using the cross-trainer, the protocol consisted of 2 min at 100 W, followed by 5–10 min at 100 W and a further 5–10 min at 100 W, with recovery intervals between exercise periods. For younger or fitter patients, the protocol consisted of 5 min at 100 W, followed by 10 min at 150 W and a further 5–10 min at 100 W.

During recovery intervals, patients were seated for approximately 2 min. Respiratory rate and facial appearance were assessed, and the Borg rating of perceived exertion was assessed when clinically appropriate. Vital parameters were reassessed during these intervals. If the patient tolerated the exercise well, the duration of the exercise intervals could be increased or the workload increased. For older or untrained patients, workload was increased by approximately 10–20 W; for younger or fitter patients, increases of approximately 25 W were used.

The intervention protocol did not specify a single numerical Borg target or predefined numerical stopping thresholds. Exercise intensity and progression were therefore individualized according to clinical tolerance and the patient's postoperative condition. The sessions were conducted in the exercise room on the postoperative ward by the doctoral researcher under the supervision of a physiotherapist. Each session lasted a maximum of 30 min (Suppl. Tbl. 1).

Following discharge, patients received instructions for continued self-training.

On postoperative day 3 all groups completed the Quality of Life Questionnaire (QLQ-C30). After 30 days, all groups were assessed using the Questionnaire Quality of Recovery (QoR-15).

### Primary outcomes

Feasibility was assessed descriptively using recruitment, retention, and intervention adherence as prespecified feasibility indicators. Recruitment was expressed as the proportion of screened patients who were randomized. Retention was defined as the proportion of randomized patients who completed the study. Intervention adherence was defined as completion of all three scheduled supervised exercise sessions by participants allocated to the intervention group. No a priori thresholds for recruitment, retention, or adherence were defined, as this pilot study was primarily intended to assess the practical feasibility of the intervention and to inform the design of a future adequately powered trial.

Safety was assessed prospectively throughout the supervised exercise sessions by monitoring for intervention-related adverse events, including hemodynamic instability, excessive fatigue, exacerbation of pain, or other clinically relevant deterioration during or immediately after exercise. Any adverse event judged by the study team to be temporally and clinically related to the exercise intervention was considered an intervention-related adverse event.

The originally registered primary endpoint was time to first bowel movement. During study conduct, the principal investigator revised the outcome hierarchy and designated safety and feasibility as the primary outcomes before completion of recruitment and before examination of outcome data. This modification concerned the outcome hierarchy only and did not alter study procedures, intervention delivery, risk profile, or eligibility criteria. The change was documented internally within the study records. Time to first bowel movement was subsequently analyzed as a secondary/exploratory endpoint.

### Secondary outcomes

Handgrip strength and the Short Physical Performance Battery (SPPB) were selected as complementary objective measures of physical performance. The SPPB assesses lower-extremity physical performance through standardized measures of gait speed, chair-stand performance, and balance and has demonstrated established validity and reliability (Guralnik et al. 1994). Handgrip strength was selected as a complementary measure of muscle strength and physical capability (Kameniar et al. 2024; Tantisarasart et al. 2026; Mazzini and Leandro-Merhi 2026). Handgrip strength was assessed in both hands using a hand dynamometer (Kern & Sohn GmbH, Handkraftgerät, Balingen-Frommern, Germany) according to a standardized measurement protocol, with three trials performed per side. Because measurements were obtained during the immediate postoperative period, changes were interpreted cautiously and were not considered a direct measure of changes in muscle mass.

The SPPB was performed according to the standardized protocol. Perceived exertion was assessed using the Borg rating of perceived exertion scale (Borg and Linderholm 1970). Surgical complications were assessed on postoperative days 1 and 3 and at 30 days using the Clavien–Dindo classification (Dindo et al. 2004).

Quality of life was measured by the QLQ-C30 (Aaronson et al. 1993). Quality of recovery was measured by the QoR-15 (Myles et al. 2022). Both questionnaires are validated.

Time to first bowel movement was recorded in minutes from the end of surgery. Patients were asked regularly about bowel function and received solid food according to the local ERAS protocol. A bowel movement was defined as passage of stool according to the Bristol Stool Form Scale; passage of flatus alone was not considered a bowel movement.

Postoperative ileus was assessed separately from time to first bowel movement. Because no universally accepted definition of postoperative ileus exists, the study followed a clinical assessment based on the presence of gastrointestinal dysfunction, including absence of stool or flatus, intolerance of oral intake, abdominal distension, nausea or vomiting, and the need for nasogastric decompression or other treatment for ileus. The definition was based on previously proposed standardized criteria for postoperative ileus (Vather et al. 2013).

### Sample size calculation

Given the limited evidence base, this study was designed as a pilot trial primarily aimed at generating preliminary estimates of safety and feasibility to inform future adequately powered confirmatory trials. According to the local policies of the Ethics Committee, pilot studies should be conducted with 15 patients per arm.

### Statistical analysis

All analyses were performed on a modified intention-to-treat basis. One patient withdrew consent after randomization but before surgery and was therefore excluded from the final analysis because no postoperative study data were available.

Continuous variables are presented as mean ± standard deviation (SD) and range, as appropriate. Categorical variables are summarized as absolute and relative frequencies.

Given the exploratory nature of this pilot study and the limited sample size, no formal sample size calculation was performed. Distributional assumptions for continuous variables were assessed using graphical methods, including histograms and Q–Q plots. Based on these assessments, and to ensure robustness of inference, both parametric and non-parametric statistical approaches were applied where appropriate.

For comparisons of continuous variables between groups, independent samples t-tests were used when distributional assumptions were considered acceptable. In cases of potential deviations from normality or for sensitivity analysis, Wilcoxon rank-sum tests (Mann–Whitney U test) were additionally performed. Homogeneity of variances was evaluated using the Folded F test, and t-test results were reported using either pooled or Satterthwaite-adjusted degrees of freedom as appropriate.

Categorical variables were compared using Pearson’s chi-square test or Fisher’s exact test, as appropriate based on expected cell counts. For ordinal or non-normally distributed continuous outcomes, Kruskal–Wallis or Wilcoxon rank-sum tests were applied as indicated.

All statistical tests were two-sided, and a *p*-value < 0.05 was considered statistically significant. Given the exploratory nature of the study, no adjustments for multiple comparisons were performed. All analyses were conducted using R version 4.5.3.

### CONSORT compliance statement

The study was conducted and reported in accordance with the Consolidated Standards of Reporting Trials (CONSORT) guidelines for randomized controlled trials. A completed CONSORT checklist has been provided as supplementary material to ensure transparent and comprehensive reporting of all essential trial design, conduct, and analysis elements.

The trial was retrospectively registered in the German Clinical Trials Register (DRKS), and the full study protocol is publicly accessible via the registry entry.

Randomization was performed using a computer-generated block randomization scheme, and allocation concealment was ensured prior to patient enrollment as described in the Methods section. One patient withdrew consent after randomization and prior to intervention; accordingly, a modified intention-to-treat approach was applied.

All prespecified outcomes were reported, and any deviations from the registered protocol have been transparently disclosed in the manuscript. The reporting adheres to CONSORT recommendations for pilot and feasibility randomized trials, with particular emphasis on transparency of patient flow, feasibility metrics, and outcome definitions.

## Results

A total of 72 patients were initially screened between November 2024 and September 2025. Of these, a total of 31 patients ((Interventional group/control group) mean [SD] age, 64.3 [13.9]/62.2 [16.6] years)) met the inclusion criteria and agreed to participate.

One patient (intervention group) dropped out (3.2% drop out rate) for personal reasons. All other patients successfully underwent either intervention or routine care, completing all required assessments.

**Fig. 1:**
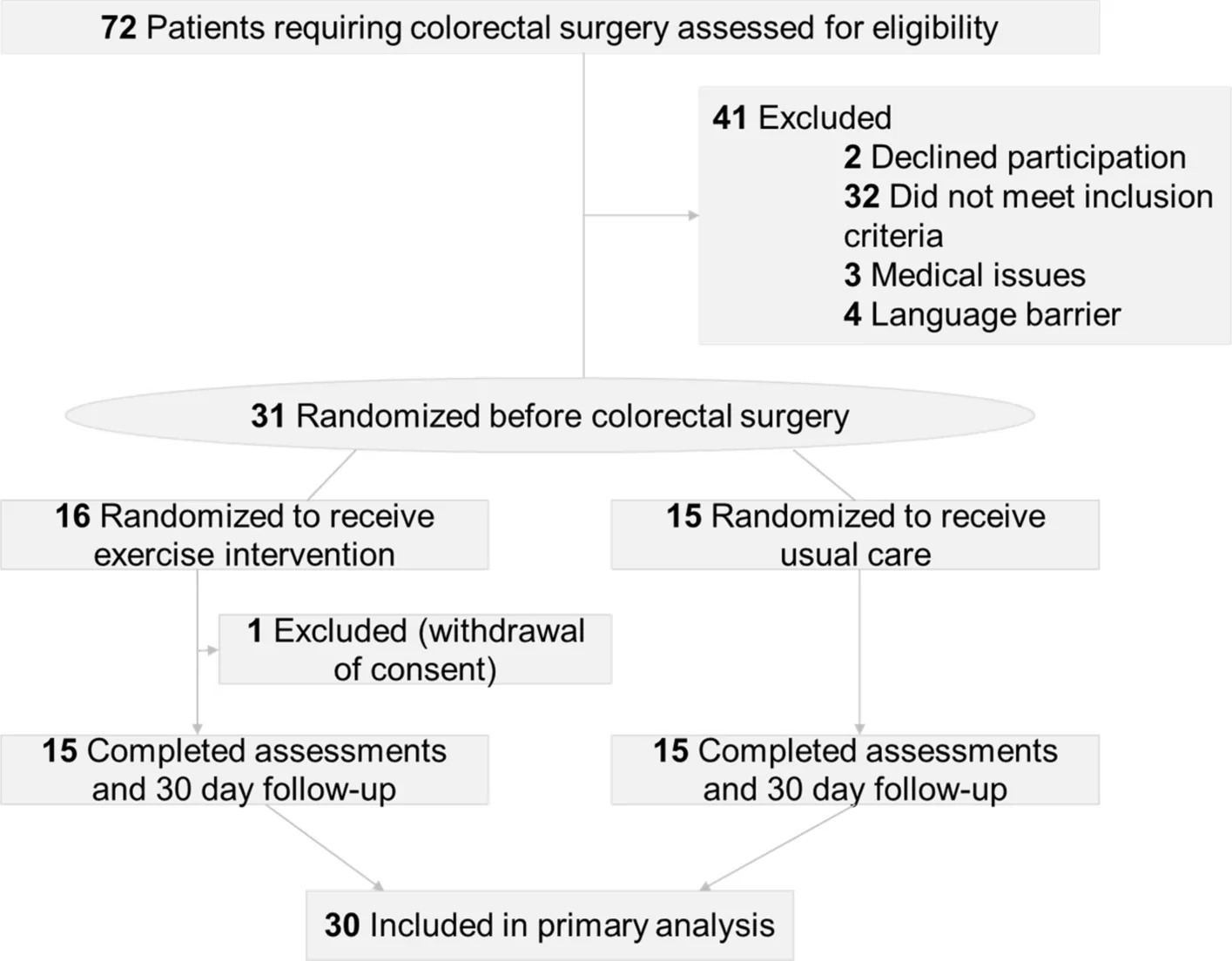
Flow diagram of screened and included patients

Figure 1 shows a flow diagram of all patients. 72 adults of any sex were screened. 41 patients were excluded due to not meeting the inclusion criteria, due to medical issues, refusal of consent, or language barrier. Feasibility outcomes are summarized in the study flow diagram. Recruitment, retention, and adherence rates demonstrated that implementation of the supervised postoperative exercise intervention was feasible within the pilot study setting. No formal acceptability assessment was performed. Full adherence to the intervention protocol was observed, with all patients in the intervention group completing all scheduled training sessions (adherence rate: 100%).

Table 1 shows patient characteristics.

**Table 1:** Basic patient characteristics (*n* = 15 per group)

**Characteristic**	**Exercise (*n* = 15)**	**Usual care (*n* = 15)**
Basic preoperative data		
Age, mean (SD), y	64.3 (13.9)	62.2 (16.9)
Weight, mean (SD), kg	76.3 (16.6)	77.5 (13.3)
BMI, mean (SD)	27.1 (4.9)	26.8 (4.2)
ECOG	0	0
ASA	2	3
Anticoagulation preop	3 (20%)	2 (13%)
CIBD (%)	0 (0%)	1 (6.7%)
Diabetes (%)	2 (13%)	1 (6.7%)
Radiation preop. (%)	2 (13%)	3 (20%)
Chemo preop. (%)	5 (33%)	5 (33%)
Immunocompromised preop. (%)	1 (6.7%)	1 (6.7%)
Intraoperative data		
Surgery duration (SD), min	283.7 (220.7)	246.4 (95.9)
Approach – open (%)	0	1 (6.6%)
Approach – laparoscopic (%)	10 (66%)	12 (80%)
Approach – robotic (%)	5 (33%)	2 (13%)
Blood loss (SD), ml	200.00 (145.8)	372.2 (410.1)
Intraoperative transfusion	0 (0%)	0 (0%)
Drain	0 (0%)	2 (13%)
PONV prophylaxis	15 (100%)	15 (100%)
Malignancy colon (%)	9 (60%)	5 (33.3%)
Malignancy rectum (%)	4 (26.7%)	5 (33.3%)
Histopathology		
Benign pathology, n (%)	2 (13.3%)	5 (33.3%)
CRC, n (%)	13 (86.7%)	10 (66.7%)
pT1 (%)	1 (7.7%)	0 (0%)
pT2 (%)	6 (46.2%)	2 (20%)
pT3 (%)	5 (38.5%)	5 (50%)
pT4 (%)	1 (7.7%)	3 (30%)
N0 (%)	8 (61.5%)	5 (50%)
N1 (%)	4 (30.8%)	3 (30%)
N2 (%)	1 (7.7%)	2 (20%)
G1 (%)	0 (0%)	0 (0%)
G2 (%)	10 (76.9%)	7 (70%)
G3 (%)	2 (15.4%)	1 (10%)
G unknown (%)	1 (7.7%)	2 (20%)
R0 (%)	13 (100%)	10 (100%)

^1^*Abbreviations: BMI* body mass index (calculated as weight in kilograms divided by height in meters squared), *ECOG* Eastern Cooperative Oncology Group, *ASA* American Association of Anesthesiology, *CIBD* chronic inflammatory bowel disease, *PONV* postoperative nausea and vomiting, *CRC* colorectal cancer

### Baseline characteristics

Baseline demographic and clinical characteristics were generally comparable between groups. Mean age was 64.3 years (SD 13.9) in the intervention group and 62.2 years (SD 16.9) in the control group. BMI, ECOG performance status, ASA classification, and prevalence of comorbidities (diabetes, chronic inflammatory bowel disease, anticoagulation use) were similar across groups.

Similarly, intraoperative parameters did not differ significantly between groups. This includes surgery duration, surgical approach (open, laparoscopic, or robotic), intraoperative blood loss, the need for intraoperative transfusion, drain placement, and administration of prophylaxis for postoperative nausea and vomiting (PONV). Furthermore, the distribution of malignancy location, colon, sigmoid, or rectal, was comparable across groups, with no statistically significant differences detected.

No statistically significant differences were identified between the groups with regard to postoperative outcomes, including Clavien–Dindo classification, in-hospital mortality, and length of in-hospital stay (days).

Baseline characteristics were generally comparable between groups; however, clinically relevant imbalances may still have been present due to the small sample size.

### Primary outcome: feasibility and safety

Of 72 screened patients, 31 were randomized, corresponding to a recruitment rate of 43.1%. Thirty patients completed the study, resulting in a retention rate of 96.8%. All participants allocated to the intervention group completed the three scheduled supervised exercise sessions, corresponding to an intervention adherence of 100%.

No intervention-related adverse events were observed during or immediately after the supervised exercise sessions. Specifically, no episodes of hemodynamic instability, excessive fatigue, exacerbation of pain, or other clinically relevant deterioration attributable to the exercise intervention were recorded.

### Secondary outcomes

Secondary outcomes, such as time to first bowel movement, performance at SPPB, Borg-Scale, handgrip strength as well as Quality of Life and Recovery are listed below. Table 2 shows postoperative effects.

**Table 2:** Intervention effects on bowel movement, surgical complications, and hospital-stay among patients after elective colorectal surgery (*n* = 15 per group)

**Variable**	**Exercise (*n* = 15)**	**Usual care (*n* = 15)**	***Between-group difference (95% CI)***	***P* value**
Bowel movement, min	2584	2427	+ 157 (− 721 to 387)	0.56
SPPB, delta mean (SD)	−0.33 (2.09)	−3.07 (2.31)	− 2.74 (− 4.21 to − 1.27)	0.002
Clavien-Dindo	0	0	N/A	N/A
In-hospital-stay, d	5	5	0 (− 1 to 1)	0.87
Handgrip strength, delta mean (SD)	−0.84 (2.43)	−2.68 (5.94)	−1.84 (− 5.31 to 1.63)	0.13

#### SPPB

**Fig. 2:**
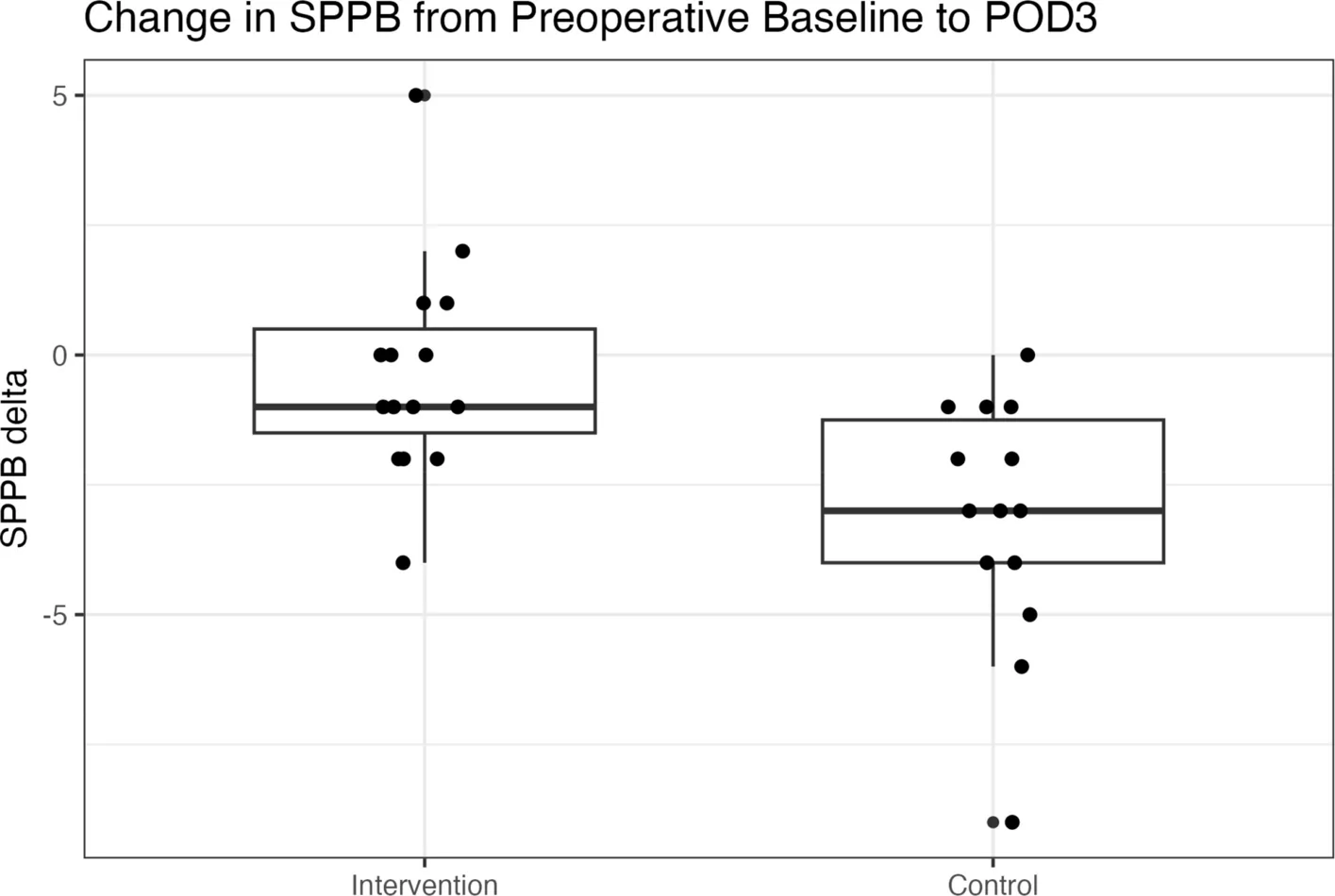
Change in SPPB over time. Patients in the intervention group showed a smaller decline in SPPB scores compared with the control group

Higher SPPB scores indicate improved physical performance. ΔSPPB was calculated as preoperative minus postoperative score; therefore, more negative values indicate a greater postoperative decline in physical performance. A statistically significant between-group difference was observed, representing the main finding of this study. Patients in the intervention group demonstrated a smaller decline in SPPB scores (mean − 0.33 ± 2.09, *n* = 15) compared with the control group (mean − 3.07 ± 2.31, *n* = 15), indicating better preservation of measured physical performance. This difference was statistically significant (Mann–Whitney U = 39.0, *p* = 0.002), corresponding to a mean between-group difference of − 2.74 points (95% CI − 4.21 to − 1.27) and a moderate standardized effect size (Cohen's d = 0.68). The observed between-group difference exceeded the commonly reported minimal clinically important difference (MCID) of 1 point for the SPPB (Fig. 2).

#### Bowel movement

Time to first bowel movement, assessed in days, hours after surgery, and minutes, did not differ significantly between groups (all *p* > 0.05, between-group difference −167 95% CI −721 to 387). Median time to bowel movement was 2584 min in the intervention group and 2427 min in the control group (*p* = 0.563). No cases of postoperative ileus occurred, and no patient required postoperative nasogastric tube placement.

#### Borg

No statistically significant differences were observed between the groups in perceived exertion as measured by the Borg Scale. The mean change in Borg score from baseline to postoperative day 3 was 1.72 (SD 1.89, *n* = 29), with a 95% confidence interval of 1.01 to 2.44 (*p* < 0.0001 for within-group change). However, the between-group difference did not reach statistical significance (*p* > 0.05).

#### Handgrip strength

**Fig. 3:**
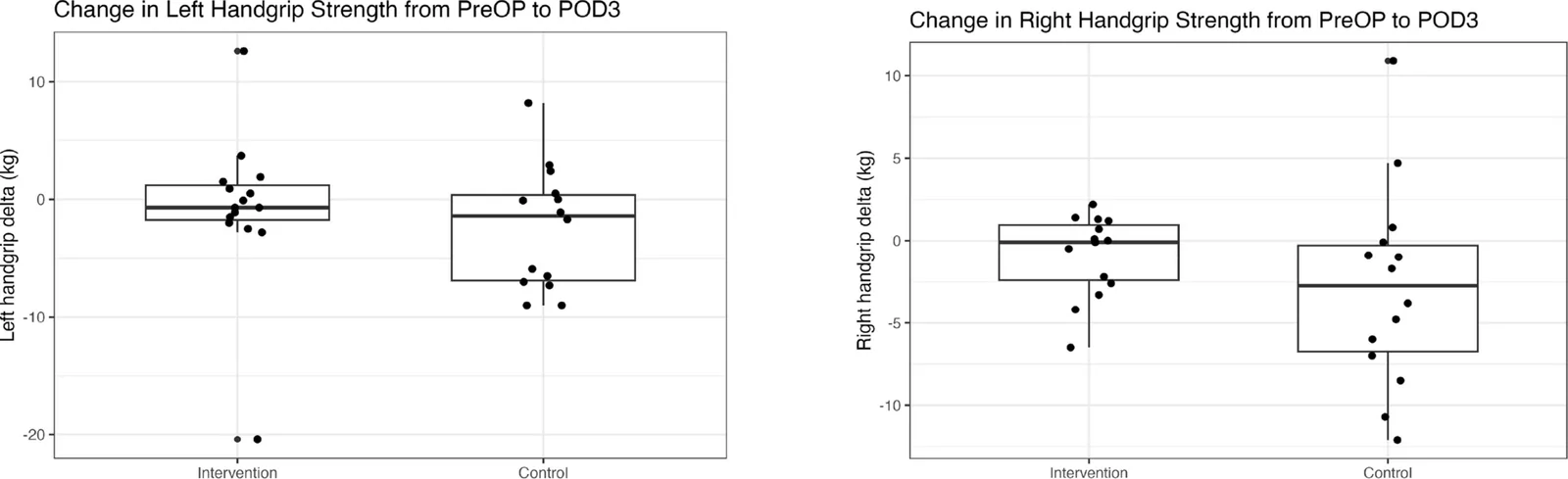
Change in handgrip strength from preoperative to postoperative day 3, differentiated in left and right hand

Handgrip strength did not differ significantly between groups (Mann–Whitney U = 75.0,* p* = 0.13, between-group difference − 1.84 CI 95% − 5.31 to 1.63). Although the intervention group showed a numerically smaller decline (mean − 0.84 ± 2.43) compared to the control group (mean − 2.68 ± 5.94), this difference did not reach statistical significance (Fig. 3).

#### Clavien-Dindo

No postoperative complications were observed in either group according to the Clavien-Dindo classification system through postoperative day 30. Specifically, no minor complications (Clavien-Dindo grade I–II, such as wound infections requiring antibiotics, urinary tract infections, or transient arrhythmias) and no major complications (Clavien-Dindo grade III–V, requiring surgical, endoscopic or radiological intervention, or resulting in permanent disability or death) occurred in either study group. The absence of complications in both groups is consistent with the low-risk nature of elective colorectal surgery within an established ERAS pathway at our institution.

#### Quality of life

No statistically significant differences were observed between the groups regarding Quality of Life outcomes as measured by the EORTC QLQ-C30 on postoperative day 3 and 30. No significant differences were detected in any of the functional scales (physical, role, emotional, cognitive, social) or symptom scales (fatigue, nausea/vomiting, pain, dyspnea, insomnia, appetite loss, constipation, diarrhea, financial difficulties).

#### Quality of recovery

Similarly, Quality of Recovery scores were comparable between the intervention and control groups. No significant differences were observed in any of the five QoR-15 dimensions (physical comfort, emotional state, physical independence, psychological support, and pain).

## Discussion

This pilot trial demonstrated that an early and individually adapted postoperative training is feasible and safe. Differences in length of hospital stay or complications according to Clavien-Dindo did not occur.

The observed difference in SPPB represents the main efficacy-related signal of this pilot trial. The intervention group showed a smaller decline in measured physical performance after surgery, and the between-group difference exceeded the commonly reported minimal clinically important difference. However, this finding should be interpreted cautiously. The study was small and was primarily designed to assess feasibility and safety rather than to establish efficacy. Moreover, the lack of prospective documentation of the fitness classification used to individualize exercise intensity means that residual confounding cannot be excluded. The observed SPPB difference therefore does not establish that the intervention improves broader postoperative recovery or leads to downstream benefits such as reduced complications, shorter hospital stay, or improved quality of life. Rather, the finding should be regarded as hypothesis-generating and warrants confirmation in adequately powered randomized trials.

Bowel movement as secondary outcome showed no statistically significant difference in both groups. Postoperative ileus affects roughly 30% of patients undergoing elective colorectal surgery (Teng et al. 2021; Abernethy and Aly 2024; Namba et al. 2021). While no cases were observed in the present study, the positive impact of physical activity on gastrointestinal recovery is well documented (Chapman et al. 2018; Elnour et al. 2025). Physical activity has been proposed as a potential contributor to postoperative gastrointestinal recovery; however, the present pilot trial did not demonstrate a measurable effect on time to first bowel movement.

The present pilot study was not designed or powered to determine whether preservation of physical performance translates into a reduction in postoperative complications. For a future confirmatory trial, the causal pathway between the intervention, functional recovery, gastrointestinal recovery, and postoperative complications should therefore be prespecified. Functional performance may serve as an intermediate outcome, while complications and other patient-centered outcomes should be evaluated as confirmatory endpoints.

Our results correlate to other studies in this area. Although, Min et al. (2023) could show positive effects on the length of the hospital stay after laparoscopic colorectal surgery, this study included open procedures, which makes a comparison difficult. Nevertheless, it is reasonable to assume that even daily exercise after both open and minimally invasive procedures may positively influence the aforementioned parameters. It should further be emphasized that supervision is essential not only for medical and safety reasons but also for ensuring adherence. This may partly explain why the study by Onerup et al. did not demonstrate significant differences in the context of an unsupervised training intervention pre- and postoperative (Onerup et al. 2022). A recent meta-analysis published in 2024 indicates that the current evidence base for both preoperative and postoperative exercise interventions remains insufficient to draw firm conclusions, in contrast to nutritional prehabilitation, where more consistent evidence has been established (Steffens et al. 2024). In particular, prehabilitation in all surgical fields has been extensively investigated in numerous trials; however, it has consistently demonstrated only weak evidence and varying outcomes, as reflected in recent studies (Onerup et al. 2022; Kamarajah et al. 2020; Barberan-Garcia et al. 2018).

No significant difference in length of hospital stay was observed between the intervention and control groups, potentially reflecting the long-standing implementation of ERAS protocols at our institution. Given the robust evidence demonstrating that ERAS pathways significantly reduce hospital stay, the potential for incremental benefits attributable to the intervention may have been limited in such small patient cohorts (Bayat et al. 2024). As previously proposed by Van der Storm, the integration of a mobile application may be feasible in the future within the framework of a structured blended training program, serving as a complementary component to the ERAS protocol and supervised in-hospital training (Storm et al. 2025).

The intervention was feasible within the infrastructure of our institution; however, its implementation in other hospitals may require additional resources. The program depended on access to suitable exercise equipment, trained personnel, physiotherapy supervision, dedicated space, and staff time for individualized sessions of up to 30 min. Therefore, feasibility in this pilot study should not be interpreted as evidence of universal implementability. Future studies should evaluate the resource requirements, staff workload, equipment availability, and cost implications of supervised postoperative exercise when assessing its transferability to routine clinical practice (Hirani et al. 2025; Markey et al. 2026).

Recent studies on colorectal anastomotic techniques and perioperative outcomes have reported improvements in short-term recovery parameters (Sun and Zhang 2024). However, post-operative recovery following colorectal surgery is multifactorial and not solely determined by adherence to ERAS protocols. In addition to standardized perioperative pathways (Gaggero et al. 2024), a variety of factors, including patient-related variables, surgical technique, anesthetic management, and perioperative complications, contribute to functional recovery trajectories. Consequently, evidence directly linking specific interventions to isolated functional recovery endpoints remains limited.

The exercise protocols across studies remain highly heterogeneous. Some interventions involved daily postoperative training, while others initiated preoperative sessions of 15 min per day. Supervision varied as well, ranging from fully supervised programs to entirely home-based regimens. Consequently, the literature demonstrates limited consistency in both protocols and outcomes. Although similar studies exist in other surgical populations, such as breast cancer or cardiac surgery, their findings are not directly transferable due to differing patient demands and procedural requirements (Westerdahl and Möller 2010; Min et al. 2024). A common feature across all protocols is that no adverse effects were reported.

Last, especially in patients unable to complete sufficient prehabilitation programs, e.g. due to time-critical surgery or limited local infrastructure, at least a close and intensified postoperative follow-up and support regimen should be ensured whenever possible (Gillis et al. 2014).

Strength and Limitations.

This study has several strengths. A major strength is the development of a structured, individualized postoperative exercise program as an adjunct to standard ERAS-based care. Exercise was adapted to individual patient characteristics and postoperative tolerance and delivered by a doctoral researcher under physiotherapist supervision. Further strengths include the randomized design, close adherence to the intervention, and standardized outcome assessment by a single assessor. The intervention was feasible within our institutional infrastructure; however, implementation required suitable equipment, trained personnel, physiotherapy supervision, dedicated space, and staff time for individualized sessions. Future studies should therefore also consider healthcare resource use and the requirements for integration into routine clinical care.

The main limitations are the single-center design and small sample size, which limit generalizability and may have resulted in clinically relevant baseline imbalances despite randomization. Consequently, residual confounding cannot be excluded, and efficacy-related findings should be interpreted as exploratory and hypothesis-generating. Patients and intervention providers could not be blinded, which may have introduced performance and detection bias. In addition, patients in the usual-care group did not receive in-person postoperative exercise instruction, which may limit comparability with settings where postoperative physiotherapy is routinely provided. Quality of life was assessed postoperatively only at day 30, and postoperative diet progression was not prospectively recorded, although all patients received solid food from postoperative day 1.

The interpretation of handgrip strength also requires caution because early postoperative measurements may be influenced by fatigue, pain, fluid shifts, residual effects of anesthesia, and the physiological stress of surgery. Furthermore, the “younger/fitter” versus “older/untrained” classification used to guide exercise intensity was not prospectively documented. Although age, ECOG performance status, and ASA classification were generally comparable between groups, differences in baseline functional capacity or exercise tolerance may not have been captured, potentially contributing to residual confounding, particularly for the SPPB outcome. Future trials should prospectively define and record baseline fitness and functional status using standardized criteria.

Finally, the outcome hierarchy was modified after trial registration, with safety and feasibility designated as the primary outcomes during study conduct. Although this change was made before completion of recruitment and before examination of outcome data and did not alter the study procedures, this post-registration modification warrants cautious interpretation. Future studies should prospectively define the outcome hierarchy and may consider incorporating additional strength and stretching components into the exercise program.

## Conclusions

Early supervised postoperative exercise following elective colorectal surgery was feasible, and no intervention-related safety concerns were observed in this pilot trial. The intervention group demonstrated better preservation of postoperative physical performance as measured by the SPPB. However, no significant differences were observed for gastrointestinal recovery, handgrip strength, quality of life, quality of recovery, postoperative complications, or length of hospital stay. Given the small sample size and exploratory nature of the study, these findings should be considered hypothesis-generating and require confirmation in adequately powered multicenter randomized trials. Future trials should evaluate whether combining aerobic and resistance exercise with structured home-based training improves postoperative functional recovery and can be integrated into routine clinical care.

## Supplementary Information

Supplementary Material 1: Supplementary Table 1. Description of the postoperative exercise intervention according to the TIDieR checklist

## Data Availability

All data supporting the findings of this study are available within the paper and its Supplementary Information.

## Abbreviations

ASA: American Society of Anesthesiologists
BMI: Body Mass Index
CIBD: Chronic Inflammatory Bowel Disease
CRC: Colorectal Cancer
ECOG: Eastern Cooperative Oncology Group performance status
ERAS: Enhanced Recovery After Surgery
kg: Kilogram
MCID: Minimal clinically important difference
min: Minute(s)
ml: Milliliter
POMGAT: Perioperatives Management bei gastrointestinalen Tumoren
PONV: Postoperative Nausea and Vomiting
POD3: Postoperative Day 3
PreOP: Preoperative
QLQ-C30: European Organisation for Research and Treatment of Cancer Quality of Life Questionnaire
QoR-15: 15-Item Quality of Recovery scale
SD: Standard Deviation
SPPB: Short Physical Performance Battery
