# Supplementary material for: Early implementation of exercise to facilitate recovery after elective colorectal surgery—a randomized clinical pilot trial
Source: Perioper Med (Lond). 2026 Oct 16;15:94. doi: 10.1186/s13741-026-00752-8 (PMC13675950; doi:10.1186/s13741-026-00752-8)
Supplement: Supplementary file 1 [file 13741_2026_752_MOESM1_ESM.docx]

Supplementary material

Supplementary Table 1. Description of the postoperative exercise intervention according to the TIDieR checklist

| **TIDieR item** | **Description** |
| --- | --- |
| **1. Brief name** | **Supervised early postoperative exercise intervention** |
| **2. Why – Rationale** | The intervention was designed as an adjunct to standard ERAS-based postoperative care to provide structured, individualized physical exercise during the early postoperative period. The aim was to support postoperative physical performance and functional recovery. |
| **3. What – Materials** | A **cycle ergometer or cross-trainer** was used for the supervised exercise sessions. No additional intervention-specific materials were specified in the original protocol. |
| **4. What – Procedures** | Patients performed structured exercise intervals on either a cycle ergometer or cross-trainer. Exercise workload and duration were individualized according to age, preoperative functional status, previous exercise experience, and postoperative clinical tolerance. Recovery intervals of approximately 2 min were provided between exercise periods. During recovery, patients were seated and clinical tolerance was assessed. |
| **5. Who provided** | The intervention was **performed by the doctoral researcher under the supervision of a physiotherapist**. |
| **6. How** | The intervention was delivered **face-to-face and individually** to each patient. |
| **7. Where** | All intervention sessions were conducted on the ward in a specialized exercise area. |
| **8. When and how much** | Sessions were performed on **postoperative days 1, 3, and 5**. Each session lasted a **maximum of 30 min**, including exercise and recovery intervals. |
| **9. Tailoring** | Exercise was individualized according to patient age, preoperative fitness/functional status, previous exercise experience, and postoperative clinical tolerance. Patients were broadly categorized as **older/untrained beginners** or **younger/fitter patients**. Workload and/or exercise duration could be increased when exercise was well tolerated. |
| **10. Modifications** | For older/untrained patients, workload could be increased by approximately **10–20 W**. For younger/fitter patients, workload could be increased by approximately **25 W**. Exercise intervals could also be prolonged according to tolerance. |
| **11. How well – Planned adherence/fidelity** | The protocol specified supervised sessions on postoperative days 1, 3, and 5. Intervention adherence was assessed by documenting whether the scheduled exercise sessions were completed. **No formal fidelity assessment beyond intervention adherence was specified.** |
| **12. How well – Actual adherence/fidelity** | Intervention adherence was 100%. |

| **Patient category** | **Exercise modality** | **Initial exercise protocol** | **Progression** |
| --- | --- | --- | --- |
| **Older / untrained beginners** | Cycle ergometer | 5 min at 25 W → recovery → 5–10 min at 50 W → recovery → 5 min at 25 W | If well tolerated, exercise intervals could be prolonged or workload increased by approximately 10–20 W |
| **Older / untrained beginners** | Cross-trainer | 2 min at 100 W → recovery → 5–10 min at 100 W → recovery → 5–10 min at 100 W | If well tolerated, exercise intervals could be prolonged or workload increased by approximately 10–20 W |
| **Younger / fitter patients** | Cycle ergometer | 5 min at 50 W → recovery → 5–10 min at 75–100 W → recovery → 5 min at 50 W | If well tolerated, exercise intervals could be prolonged or workload increased by approximately 25 W |
| **Younger / fitter patients** | Cross-trainer | 5 min at 100 W → recovery → 10 min at 150 W → recovery → 5–10 min at 100 W | If well tolerated, exercise intervals could be prolonged or workload increased by approximately 25 W |
